# Biosourced Binder for Wood Particleboards Based on Spent Sulfite Liquor and Wheat Flour

**DOI:** 10.3390/polym10101070

**Published:** 2018-09-27

**Authors:** Ana M. Ferreira, João Pereira, Margarida Almeida, João Ferra, Nádia Paiva, Jorge Martins, Fernão D. Magalhães, Luísa H. Carvalho

**Affiliations:** 1 LEPABE—Faculdade de Engenharia da Universidade do Porto, Rua Dr. Roberto Frias, 4200-465 Porto, Portugal; amf@fe.up.pt (A.M.F.); malmeida@fe.up.pt (M.A.); jmmartins@demad.estv.ipv.pt (J.M.); fdmagalh@fe.up.pt (F.D.M.); 2 ARCP—Associação Rede de Competência em Polímeros, Rua Júlio de Matos, 828 4200-455 Porto, Portugal; jamp@fe.up.pt; 3 Euroresinas—Industrias Químicas S.A., Plataforma Industrial de Sines, 7520-064 Sines, Portugal; joao.ferra@sonaearauco.com (J.F.); nadia.paiva@sonaearauco.com (N.P.); 4 DEMad—Departamento de Engenharia de Madeiras, Instituto Politécnico de Viseu, Campus Politécnico de Repeses, 3504-510 Viseu, Portugal

**Keywords:** bioadhesives, wood composites, spent sulfite liquor, wheat flour

## Abstract

Currently, the majority of binders used in wood particleboard (PB) manufacturing are formaldehyde-based synthetic resins. Because of the toxicity of formaldehyde, there is a strong demand for eco-friendly alternatives with similar performances and economic viability. In this work, thick spent sulfite liquor (TSSL), an industrial byproduct from sulfite pulp mills, is proposed as a binder for fully bio-based PBs. The results showed that PBs bound with TSSL present appropriate mechanical performance, which was further improved when TSSL was combined with wheat flour at an 84:16 dry weight ratio and preheated to 94 °C prior to application. For 13.2% binder content per dry wood weight, the PB internal bond strength was 0.46 N mm^−2^, which is above the standard requirements for PB type P2 (0.35 N mm^−2^). Optical microscopy showed that TSSL hinders the gelatinization of starch granules during preheating, allowing the binder mixture to maintain a low viscosity suitable for combination with wood particles and PB production.

## 1. Introduction

Particleboards (PBs) are wood-based panels formed from small wood particles bonded with an adhesive and pressed together at high temperatures. Europe produces approximately 29 million m^3^ of PBs per year, and this value is expected to continue to grow [1]. Currently, the majority of adhesives used in PB manufacturing are synthetic and mainly formaldehyde-based because of their high reactivity, good binding strength, and low cost [2]. Nevertheless, these resins release a small amount of formaldehyde, a toxic chemical compound obtained from non-renewable resources and classified as a human carcinogen [3], which raises public concerns.

Society has become more aware of environmental and human health concerns, which has created a strong demand for sustainable products, such as natural wood adhesives. In Europe, the bio-economy is worth over 2.2 trillion euros, responsible for 18 million jobs, and has great potential to create competitiveness and add value in many sectors [4]. Wood adhesive producers have been looking for eco-friendly and economically viable alternatives by considering the availability, cost, and competition for food and energy of natural resources. Additionally, other factors must be considered, such as the ease of formulation, application, and the impact on the physical-mechanical performance of PBs [5].

Bio-based adhesives for wood composites have been the subject of several reviews [6,7,8,9,10]. The most studied natural products have been tannins, soy protein, and lignin. Usually these compounds are combined with synthetic resins or cross-linkers, as epichorohydrin and isocyanates, to improve their properties. PBs manufacture with natural adhesives usually requires longer pressing times (7.5 min to 20 min) and higher binder contents, which can go up to 30 wt%, than when synthetic, highly reactive, resins are used. The boards density ranges from 650 kg·m^−3^ to 1000 kg·m^−3^ and the pressing temperature from 180 °C to 220 °C. As the PBs bonded with natural adhesives would require longer production times, the raw materials must be cost-effective to balance manufacture costs.

Spent sulfite liquor (SSL), also known as brown liquor, is a byproduct from the acidic sulfite wood pulping process and is burned for energy production in most pulp mills [11] because of lack of applications. This liquor is usually concentrated using multiple evaporators, which results in thick spent sulfite liquor (TSSL) with slightly lower contents of carbohydrates, furfural, and acetic acid [12]. Marques et al. [12] analyzed the chemical composition of TSSL from acidic magnesium-based sulfite pulping of *Eucalyptus globulus* and determined that the major components are lignosulfonate lignin (58% of TSSL dry solids), ashes (24% of TSSL dry solids), and sugars (16% of TSSL dry solids). Lignosulfonate lignin is generated from the incorporation of sulfonate groups (SO_3_^−^) into the benzylic carbon of the phenylpropane units of lignin during the sulfite pulping process, which makes it an anionic polyelectrolyte that is extremely soluble in water [13] and consequently extractable from cellulose.

The dominant sulfite pulping process in Europe is magnesium sulfite pulping [14] and among all of the technical lignins, lignosulfonates (LS) are the most commercially available and most exploited for different applications [13]. These applications include binders, flocculants, dispersing agents, and concrete water reducers [15,16]. Technical lignin contains hydroxyl groups (phenolic and aliphatic), free ortho positions on the phenolic ring, and, in lower amounts, carboxyl or carbonyl groups. These functional groups enable not only the application of lignin in the production of polymeric material, but also chemical modification [13].

Despite LS being more useful as a raw material, TSSL is more economically compelling. The first patents that mentioned the application of SSL as an adhesive for paper, wood, and lignocellulosic materials date from the end of the 19th century [17]. Over the next several years, various studies worked on the development of an adhesive made solely with SSL. In 1962, the Pederson process was developed, consisting of producing wood composites with SSL by autoclaving pressed PBs for 2 h to improve the bonding properties. Despite the good performance of the manufactured PBs, the production costs and environmental problems made this process less attractive [18]. In 1972, a process was patented for making adhesives from SSL by treating it with an oxidant and catalyst to induce radical polymerization. The industrial application of this patent created some problems in wood panel plants, such as machinery corrosion, because of hydrogen peroxide, and handling problems, as the boards were relatively soft after being pressed [19]. In 1980, the polymerization of lignin using an enzyme (phenol oxidase) was studied to bond wood [18]. This approach is still being researched, but the cost of implementation still makes it unfeasible [20].

Even though there are several works in the literature regarding the combination of SSL with synthetic resins, such as phenol-formaldehyde and urea-formaldehyde, the industrial-scale feasibility of the reported approaches is debatable [21]. On the other hand, there is little information concerning adhesive performance of TSSL, alone or combined with other natural compounds. In the present work, TSSL was tested as an adhesive for particleboards and subsequently combined with wheat flour to obtain an eco-friendly and low-cost bio-adhesive. Wheat flour is a low-cost natural product that is obtained from wheat grains and consists mainly of starch (72.8%) and proteins (7.8%) [22]. Wheat flour is widely used as an extender for synthetic resins in wood products [23] and its main component (starch) is known for its good adhesive performance, as it is able to interact with wood surfaces after gelatinization of starch granules [8]. Proteins (gluten) in wheat flour (WF) also present adhesive properties because glutenin and gliadin crosslink when subjected to heat [24], and its wood adhesion properties have been widely studied [25,26,27,28,29].

## 2. Materials and Methods

### 2.1. Materials

Thick spent sulfite liquor (53 wt % to 59 wt % solids content) from the acidic magnesium-based sulfite pulping process of *Eucalyptus globulus* was supplied by Caima-Indústria de Celulose SA (Constância, Portugal). The WF was a 55 type commercial product obtained from Pingo Doce (Lisboa, Portugal) with the following composition: 76.2% carbohydrates, 12.5% water, 10.0% proteins, and 1.3% lipids. Wood particles and beech veneer were supplied by Sonae Arauco Portugal, SA (Oliveira do Hospital, Portugal). The wood particles were a mixture of around 30% maritime pine, 15% eucalypt, 25% pine sawdust, and 30% of recycled wood. The particles had a moisture content between 3% and 4%. The size distribution for the core layer was: 6.0% > 4 mm, 50.7% > 2 mm, 33.9% > 1 mm, 7.7% > 500 µm, 1.2% > 250 µm, and 0.6% of ultralight particles. The particles of the surface layers had sizes lower than 1 mm. Unless stated otherwise, all of the other chemicals were provided by Euroresinas-Indústrias Químicas, S.A. (Sines, Portugal).

### 2.2. Thick Spent Sulfite Liquor and Wheat Flour Blend

In a jacketed glass reactor vessel equipped with a condenser, heating thermostatic bath circulator (ED (V.2), Julabo, Seelbach, Germany), and overhead stirrer, 84 parts of TSSL were blended with 16 parts of WF (solids/solids). The 84:16 ratio was used because higher WF contents increase the mixture viscosity and impair its use as an adhesive for PBs. Then, the mixture was stirred at room temperature or 94 °C for 70 min. The final solids content was adjusted to approximately 57% using deionized water.

### 2.3. Characterization of the Resins

Viscosity: The viscosity (expressed in mPa·s) was measured on a Brookfield DV-II+ Programmable Viscometer (Brookfield AMETEK, Milddleboro, MA, USA), with a LV-2(62) spindle, at 60 rotations per minute, and 25 °C.

pH: The pH values were measured with an InLab Routine Pro combined pH glass electrode with an integrated temperature probe (Mettler Toledo, Greifensee, Switzerland).

Solids content: The solids content (%) was determined by oven-drying 2 g of resin for 3 h at 120 °C. The weights of the resins before and after drying were measured. For each determination, three replicates were prepared.

FTIR: To obtain the Fourier transform infrared (FTIR) spectra, the TSSL, WF, and resins were cured at 100 °C until a constant weight was reached. Then, the formed resin films were powdered in an agate mortar. The FTIR spectra of the powdered resins was obtained using a VERTEX 70 FTIR spectrometer (BRUKER, Ettlingen, Germany) in the absorbance mode with a high sensitivity DLaTGS detector at room temperature. The spectra were recorded from 4000 cm^−1^ to 500 cm^−1^ with a resolution of 4 cm^−1^.

Microscopy: The TSSL, WF dispersed in distilled water (20.2%), and TSSL+WF mixtures were analyzed directly at a magnification of 1000× with an optical microscope equipped with a MU500 Digital Camera (AmScope, Irvine, CA, USA).

### 2.4. Rheological Characterization

The pasting profiles of TSSL+WF mixture and WF dispersed in distilled water (20.2%, same wheat flour/water ratio of the binder), were analyzed by running a rotational test in an MCR 92 Rheometer (Anton Paar GmbH, Graz, Austria) using parallel plates with a diameter of 50 mm and a gap of 0.5 mm. The pasting analysis started with a pre-shear of 1 min, where the temperature was raised until 50 °C. After the pre-shear, the temperature was maintained at 50 °C for 1 min, followed by a linear ramp temperature from 50 °C to 90 °C, for 4 min long. After this step, the temperature was held for 1 min at 90 °C, and followed by a cooling linear ramp from 90 °C to 50 °C, 4 min long. During the analysis, the shear rate was kept at 200 s^−1^.

### 2.5. Dynamic Mechanical Thermal Analysis

The TSSL+WF-94 binder and a standard thermoset urea-formaldehyde (UF) resin were tested on a DMA 242 E Artemis (Netzsch, Selb, Wunsiedel, Germany) apparatus. Sample preparation was based on procedures described in the literature [30,31]. Two wood veneer strips of *Fagus sylvatica* 0.5 mm thick, 20 mm wide, and 117 mm long, were glued together with the binders (63 g/m^2^) and pressed at 105 °C for 5 min. The samples were then cut 50 mm long and 11 mm wide for testing in non-isothermal mode, 0 °C to 100 °C, at a heating rate of 2 K/min in three-point bending. Gaseous nitrogen was used to keep a controlled atmosphere during the cooling and heating steps. Samples were tested at a frequency of 10Hz with a maximum dynamic force of 10 N and a maximum sample amplitude of 60 µm. Three different runs were performed for each resin.

### 2.6. Automated Bonding Evaluation System

The automated bonding evaluation system (ABES) evaluates the bonding performance of an adhesive with a wood substrate [32,33]. Adhesive bonds are formed in a system that controls the temperature, press loading, and pressing time. After a bond is cured, the bond performance is assessed by testing the wood/adhesive samples in the shear mode [34].

To evaluate the wood bonding performance of the different resins that were produced, an ABES testing machine (Mark ABES II, Adhesive Evaluation Systems, Inc., Corvallis, OR, USA) was used. Briefly, two wood veneer strips (*Fagus sylvatica*), which were 0.5 mm thick, 20 mm wide, 117 mm long, and stored at a controlled temperature (25 °C) and relative humidity (65%), were glued together. The resin was applied manually with a spatula (6 mg over 100 mm^2^). After the desired temperature was reached (105 °C), the strips were mounted on the system with an overlapping area of 20 mm × 5 mm and pressed together at 1.2 N·mm^−2^ for different times that ranged from 30 s to 600 s. For each pressing time, three specimens were tested. After the pressing time elapsed, the edges of the strips were pulled. A standard loading rate was used (1 kN/s) and the bond strength was tested in the shear mode. The system was digitally controlled and pneumatically driven.

### 2.7. Particleboard Production

The PBs were produced by spraying the binder (TSSL, TSSL+WF or WF dispersion) formulation onto wood chips (2% to 3.5% moisture content) in a built in house laboratory glue blender. The resin solids content on the dry wood chips ranged from 7.8% to 24.0% and the moisture of the blended particles with the binder ranged from 7% to 12%, respectively. After blending, a three-layer particle mat was hand formed in a flexible aluminum container (220 mm × 220 mm × 80 mm). The percentage of wood particles per layer was 20% in the upper surface layer, 62% in the core layer, and 18% in the bottom surface layer. The final PB density was 650 kg/m^3^ ± 20 kg/m^3^. The hand-formed mats were pressed using a built in house parallel plate hot-press and set to simulate a typical PB continuous pressing operation. The pressing times were 300 s, 420 s, 600 s, 900 s, and 1500 s, while the pressing temperature was 190 °C. The final thickness of the panels was 15 mm. Three panels were produced for each pressing time.

### 2.8. Particleboard Characterization

The panels were stored in a conditioned room (20 °C and 65% relative humidity) after pressing, and the density, moisture content, internal bond (IB) strength, and thickness swelling were tested according to the European Standards EN 323 (1993), EN 322 (1993), EN 319 (1993), and EN 317 (1993), respectively. For each experiment, three replicates were used.

## 3. Results and Discussion

### 3.1. TSSL

Only a few studies exist in the literature concerning the direct use of TSSL as a binder for PBs. In the current work, two parameters were varied for PB production, the binder solids percentage relative to the dry wood particles (between 7.8% and 24.0%) and pressing time (between 300 s and 1500 s).

**Figure 1:**
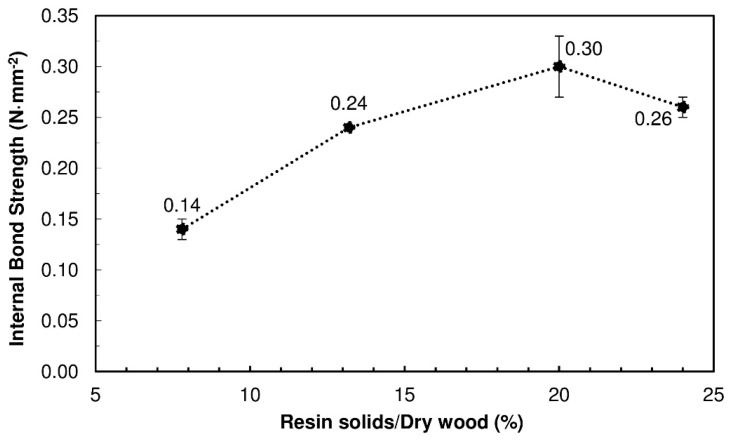
Internal bond (IB) strength of the wood particleboards (PBs) produced with thick spent sulfite liquor (TSSL) at different binder solids percentages (7.8%, 13.2%, 20.0%, and 24.0%); the PBs were manufactured at a pressing temperature of 190 °C and pressed for 600 s.

Figure 1 presents the IB strength for the produced PBs. When the content of TSSL was up to 20% binder solids, the IB strength increased proportionally with the TSSL percentage, as was expected with any binder. However, at 24% binder solids, the IB strength decreased. This was probably due to the higher moisture content in the PB, which increased the internal steam pressure during the pressing cycle. When the press opened, the pressure of the trapped steam caused springback and promoted partial breakage of adhesive bonds, resulting in mechanical strength loss.

The best result was therefore obtained with a 20% binder solids content, which produced PBs with an IB strength of 0.30 N·mm^−2^, above the requirements of the standard EN 312 (2004) for PBs type P1 (0.24 N·mm^−2^) with the same thickness (15 mm).

**Figure 2:**
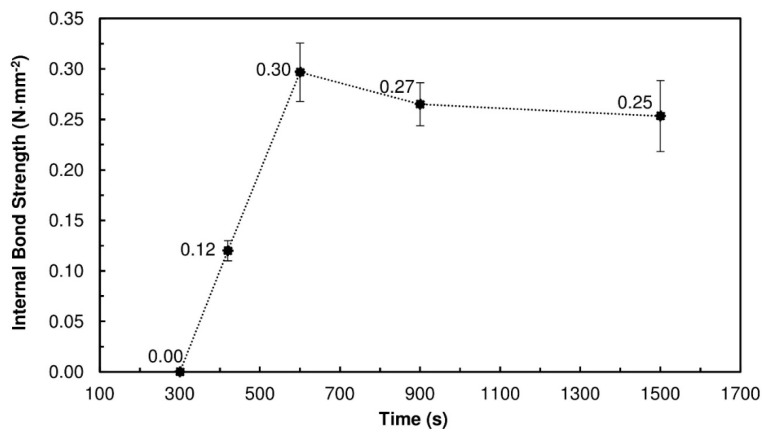
IB strength of the PBs produced with 20% TSSL at different pressing times (300 s, 420 s, 600 s, 900 s, and 1500 s); the PBs were pressed at 190 °C.

Figure 2 presents the results obtained for the PBs produced with 20% TSSL solids and pressed for different times. Between 300 s and 600 s, the IB strength increased with the pressing time, up to a maximum of 0.30 N·mm^−2^ ± 0.03 N·mm^−2^. At 300 s, the IB strength was virtually zero because of the slow thermosetting of the TSSL. Longer pressing times were needed to promote water evaporation and more interactions between the TSSL and wood. Some covalent linkages may also have been formed as the LS may cross-link under acidic pH, since TSSL contains residual furfural and sugars than can be converted to furfural during the hot pressing step [35]. Nevertheless, long pressing times are still required.

For pressing times above 600 s, the IB strength started to decrease slightly. The pressing temperature of 190 °C, combined with long pressing times (15 min and 25 min), caused degradation of the wood particles, evidenced by a burning smell and dark color of the particleboards, which had a detrimental effect on the properties of the PBs.

### 3.2. TSSL+WF Mixture

#### Basic Properties

To improve the TSSL performance, it was combined with WF, which is a low-cost product known to have good adhesive properties. Two mixtures were tested, one prepared at room temperature (TSSL+WF-RT) and the other at 94 °C (TSSL+WF-94).

The characteristics of the TSSL and TSSL+WF binders are presented in Table 1. The TSSL+WF-94 had the highest viscosity (505 mPa·s), but a substantially higher value had been expected because the mixture was prepared at a temperature higher than the gelatinization temperature of the WF starch granules, which ranges from 61 °C to 75 °C [23]. This indicated that swelling of the starch granules and leakage of amylose occurred at low degrees. Richardson et al. [36] demonstrated that LS delays the swelling of starch granules. The mechanism suggested for this effect was the molecular adsorption of LS onto the granule surfaces, which hinders transport to and from the granules. This explained why the TSSL+WF mixture did not go through gelatinization and therefore, maintained an acceptable viscosity for application on wood particles by spraying and tumble mixing. Particleboard plants usually use resins with viscosities ranging from 200 mPa·s to 600 mPa·s, to assure a uniform droplet size (the resin is atomized by either hydraulic or air spraying nozzles) and adequate distribution and penetration into the wood particles’ surface. Resin viscosity is an important factor that has a strong impact on bonding mechanical performance [37,38]. Adhesives with high viscosity do not properly wet, flow, and penetrate into the wood substrate, while adhesives with very low viscosities have excessive penetration that can lead to starved bond-lines and undesirable water absorption [37].

### 3.3. Microscopy of Flour Starch Granules

**Figure 3:**
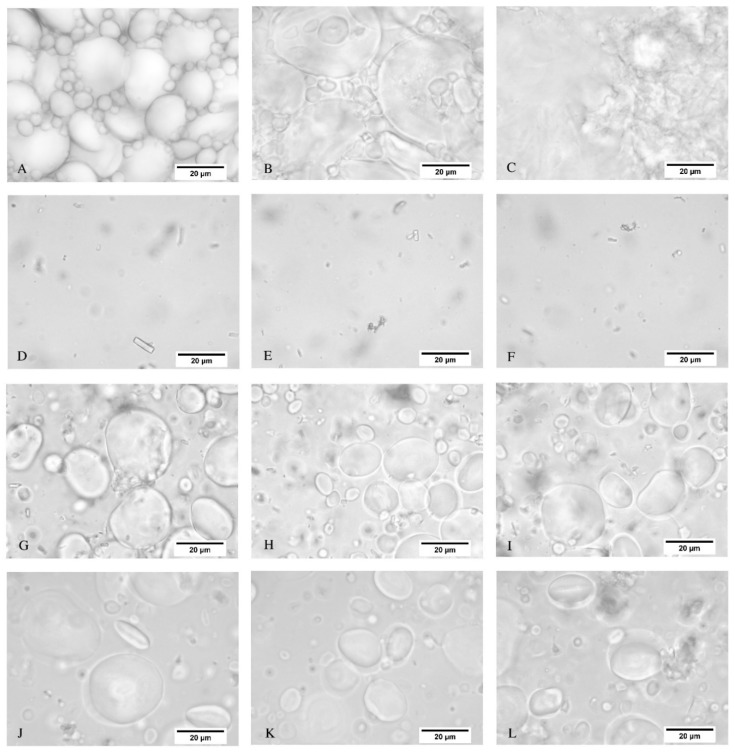
Light microscopy images of the wheat flour (WF) dispersion at room temperature (**A**) and after being heated for 2 min (**B**), and for about 15 min at 94 °C (**C**); TSSL at room temperature (**D**–**F**); TSSL+WF-RT binder at room temperature (**G**–**I**); and TSSL+WF-94 binder at room temperature (**J**–**L**). Scale bar: 20 µm.

Figure 3 depicts the optical microscopy images for the WF dispersion, TSSL, TSSL+WF-RT, and TSSL+WF-94. Figure 3A–C show that morphological changes of the starch granules of the WF were perceptible when heated at 94 °C. The granules swelled, increased in size (Figure 3B), and burst, which formed a gel when heated for a long enough time (Figure 3C).

The TSSL showed only the residues of fibers from the sulfite wood pulping process. The TSSL+WF-RT displayed starch granules dispersed in the TSSL and wood fiber residues (Figure 3G–I). Finally, the TSSL+WF-94 presented starch granules with the same morphology as that of the TSSL+WF-RT, despite having been prepared at 94 °C (Figure 3J–L). Heating did not cause perceptible changes in the granules. This result confirmed that the moderate viscosity of this resin was caused by TSSL hindering the starch gelatinization process.

### 3.4. Rheology Analysis of Flour Dispersions

**Figure 4:**
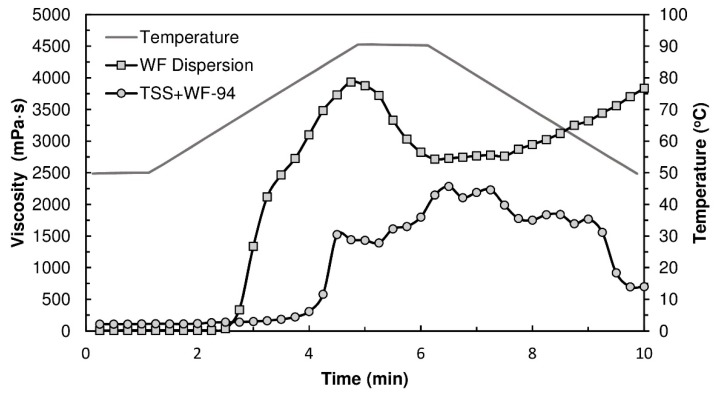
Viscograms of WF dispersion and TSS+WF-94 binder, determined with a rotational rheometer.

Figure 4 displays the viscograms of TSSL+WF-94 binder and WF dispersion. Wheat flour presents a typical starch pasting profile. After the pasting temperature (63.5 °C), the viscosity increased due to water absorption by starch granules (swelling) and leaching out of amylose chains, and a peak in viscosity was reached (3934 mPa·s). During the temperature hold stage, granule rupture occurred and the viscosity decreased. Upon cooling, the viscosity increased again due to lower molecular mobility and amylose chains re-association [39]. The TSSL+WF-94 formulation showed noticeably different rheological behavior, despite the WF/water ratio being the same. Pasting occurred at a higher temperature (77.1 °C) and was not followed by a well-defined viscosity peak, but by a region of rheological instability where viscosity oscillated around a lower value, between 1500 mPa·s and 2200 mPa·s. This indicates, as previously discussed, that the TSSL is delaying the starch gelatinization process, and the granules maintain their morphology after heating under these conditions.

### 3.5. Dynamic Mechanical Thermal Analysis

**Figure 5:**
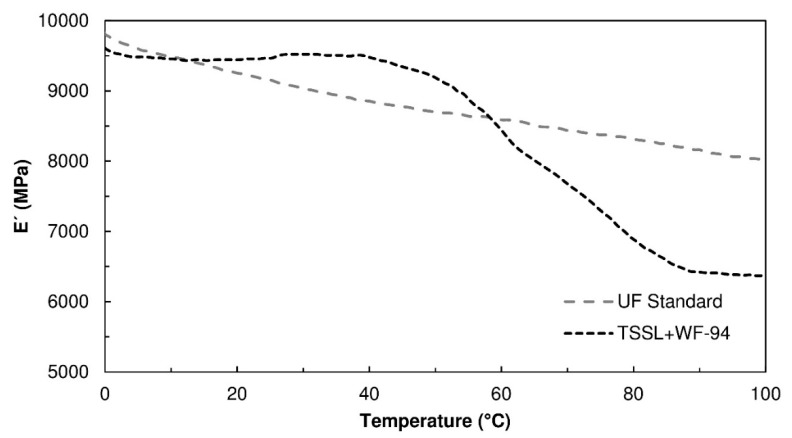
Storage modulus (*E*’) variation as a function of temperature, of beech wood joints bonded with a urea-formaldehyde (UF) standard resin and TSSL+WF-94 binder.

In order to evaluate the tendency of the TSSL-based binder to soften with temperature, i.e., the degree of temperature-dependent creep [30,31], Dynamic Mechanical Thermal Analysis (DMA) analysis was performed on a glued joint formed by two veneer strips in three-point bending mode. Figure 5 shows representative thermograms of the storage modulus, *E*’, obtained for the TSSL+WF-94 formulation, as well as for a standard urea-formaldehyde (UF) resin. The UF resin presented a slight softening with temperature increase. The reduction in storage modulus was only 18% between 0 °C and 100 °C, indicating the thermoset character of UF resins. Regarding the TSSL+WF-94 binder, the storage modulus showed a more intense decrease (33%), taking place between 40 °C and 100 °C, which may be a consequence of a thermal transition associated with some of the binder components. This can be considered a moderate degree of softening, occurring mostly at relatively high temperatures, therefore not impairing the binder’s usage for furniture production.

### 3.6. FTIR Analysis

**Figure 6:**
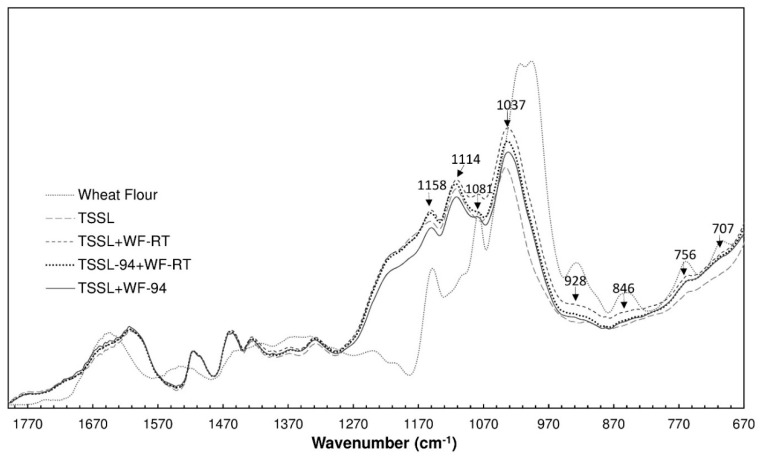
Infrared spectra of the WF, TSSL, TSSL-94+WF-RT, TSSL+WF-RT, and TSSL+WF-94.

Figure 6 presents the FTIR spectra of the dried films of TSSL, WF, TSSL+WF-RT, TSSL+WF-94, and TSSL previously heated at 94 °C for 70 min and then mixed with WF at room temperature (TSSL-94+WF-RT). The WF film spectrum absorbance is presented relative to the I_2937_ band intensity, which was assigned to C–H stretching, and the remaining spectrums using the I_1515_ band intensity, which was assigned to aromatic skeleton vibration. The spectra band assignments of the samples are given in Table 2 and Table 3.

The infrared spectra of the TSSL+WF binders were similar to the TSSL spectrum, as it is the main component (84%). Despite the similarity, the spectra presented low intensity bands characteristic of WF at different wavenumbers (1081 cm^−1^, 928 cm^−1^, 846 cm^−1^, 756 cm^−1^, and 707 cm^−1^).

The major differences among the TSSL+WF binders were observed in the bands at 1158 cm^−1^, 1114 cm^−1^, and 1037 cm^−1^. The band at 1158 cm^−1^ resulted from the overlapping of the WF band at 1149 cm^−1^ and TSSL band at 1152 cm^−1^, which were related to glycosidic linkages. The TSSL+WF-94 spectrum presented the lowest intensity at this band, which indicated that heating the TSSL+WF mixture promoted acid hydrolysis of the glycosidic bonds, which decreased their content. This occurrence was corroborated by the lower intensities of the bands at 1114 cm^−1^ and 1037 cm^−1^.

The mixture that was prepared to study the effect of heating the TSSL (TSSL-94+WF-RT) also presented a decrease in the glycosidic bonds, which indicated hydrolysis of the intermonomer bonds of the TSSL carbohydrates. These results indicated that the lower content of glycosidic bonds in the TSSL+WF-94 resulted from the hydrolysis of C–O–C bonds in the WF (primarily) and TSSL.

### 3.7. ABES Analysis

**Figure 7:**
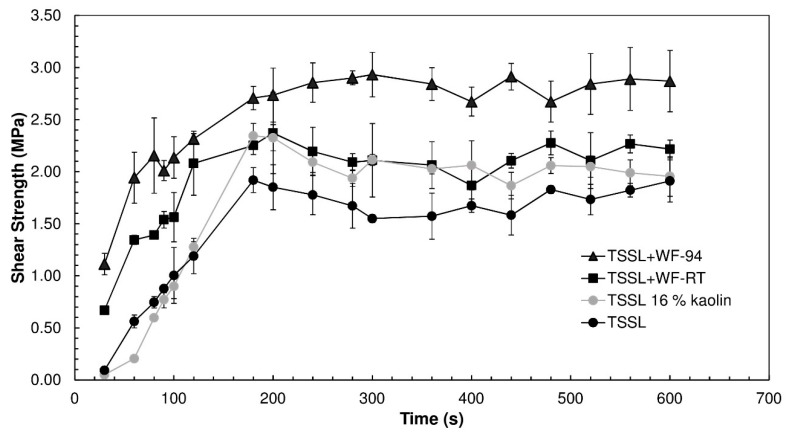
Shear strength evolution with time for the TSSL, TSSL+WF-RT, TSSL+WF-94, and TSSL 16% kaolin; the assay pressing temperature was 105 °C.

The ABES analysis was performed for an initial evaluation of the adhesive properties of the TSSL+WF formulations. Figure 7 presents the results obtained for the TSSL, TSSL+WF-RT, TSSL+WF-94, and TSSL with 16% kaolin. The latter formulation was prepared to study the effect of increasing the viscosity of the TSSL adhesive by addition of an inert filler.

It is possible to observe that incorporation of 16% WF at room temperature had a positive effect on the final formulation performance. This was promoted not only by the higher viscosity of the formulation (230 mPa·s), which controlled the degree of resin penetration into the wood, but also by the adhesive properties of the WF. This was corroborated by the results obtained from the mixture of TSSL with 16% kaolin, as it had a similar viscosity (247 mPa·s). However, because kaolin is an inert component, it did not improve TSSL performance as much as WF. Figure 7 shows that the TSSL+WF formulation presented faster curing kinetics and a slightly higher shear strength in the plateau phase. The TSSL+WF-94 binder presented better performance than the TSSL+WF-RT. This resulted of the combination of different factors, namely a higher viscosity (505 mPa·s) and lower content of glycosidic bonds, which increased the number of hydroxyl groups available to interact with wood. Moreover, starch granules pre-treated at 94 °C became more fragile, despite gelatinization being hindered by TSSL, and their structure was more susceptible to being broken in the pressing stage, facilitating interactions with the wood surface.

### 3.8. Binding Performance in the Particleboards

**Figure 8:**
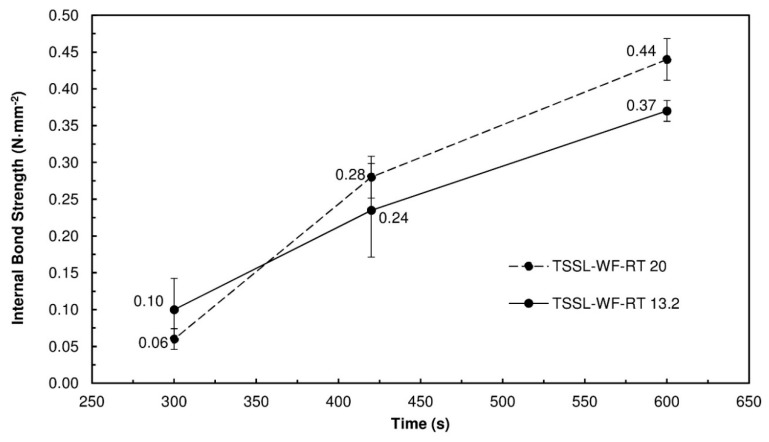
IB strength of PBs produced with the TSSL+WF-RT as a function of different pressing times; the PBs were manufactured with 20% or 13.2% binder solids relative to the dry wood particles and pressed at 190 °C.

The ABES tests indicated that the combination of TSSL with WF results in a formulation with better adhesive properties than TSSL alone. To corroborate this, PBs were produced with the TSSL+WF-RT mixture. Figure 8 presents the IB strength values obtained at different pressing times and binder/wood ratios. All of the PBs produced with the TSSL+WF formulation had a better IB strength than that of the TSSL PB, even when produced with lower resin content.

The best results were obtained with the 20% binder solids content, except at the 300 s pressing time (Figure 8). This was caused by the particle mat having a high moisture content, leading to PB delamination. Longer pressing times promoted more effective steam removal, allowing for binder hardening and increasing hydrogen bonds between binder and wood. The best results were obtained at a pressing time of 600 s, for both resin contents. At these conditions, all of the PBs produced with the TSSL+WF mixtures complied with the requirements of standard EN 312 (2004) for PB type P2 (0.35 N·mm^−2^).

**Figure 9:**
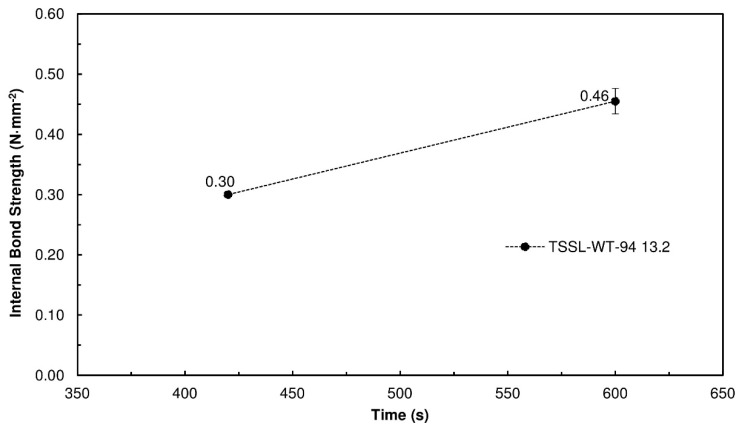
IB strength of the PBs produced with the TSSL+WF-94 as a function of different pressing times; the PBs were manufactured with 13.2% binder solids relative to the dry wood particles and pressed at 190 °C.

**Figure 10:**
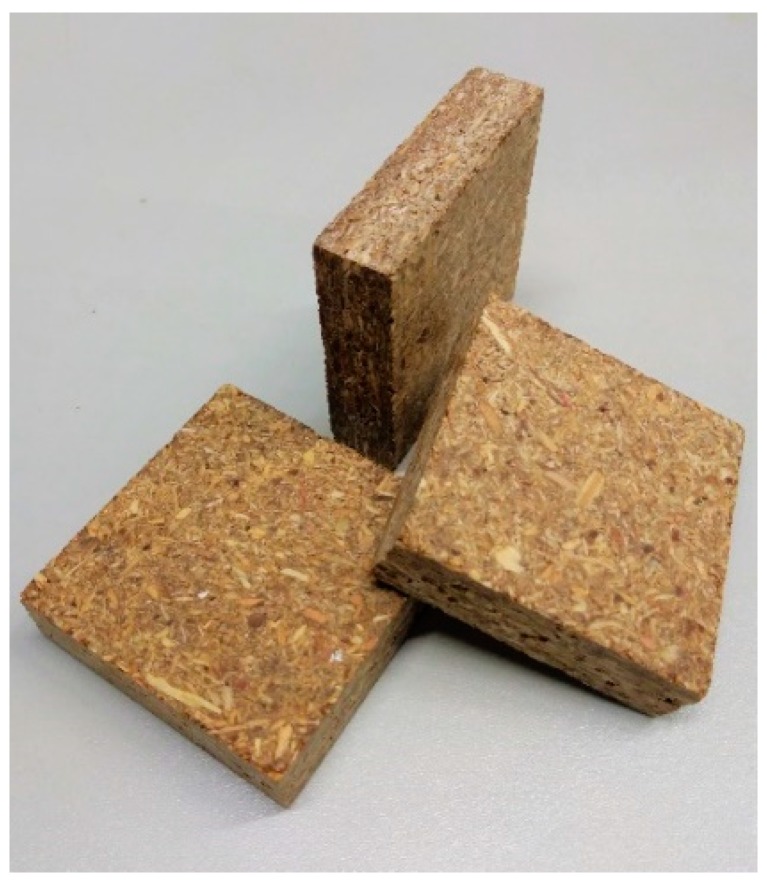
PB samples (5 cm × 5 cm) manufactured with 13.2% binder solids relative to the dry wood particles and pressed at 190 °C for 10 min.

Figure 9 presents the performance of the PBs produced with 13.2% binder content of TSSL+WF-94. Figure 10 shows the PBs that were produced.

The results showed that the mixture of TSSL with WF heated at 94 °C led to an increase in the bonding performance, which was in accordance with the results of the ABES tests. The TSSL+WF-94 yielded slightly better IB strength values at a 13.2% binder content than the TSSL+WF-RT at 20% (Figure 9).

To assess the binding contribution of TSSL in the TSSL+WF-RT formulation at 20%, new PBs were produced with the same amounts of WF and water, but without TSSL present. The panels obtained showed no cohesion (IB zero) for pressing times below 600 s, and for this pressing time, a very low IB ((0.02 ± 0.01) N·mm^−2^) was obtained. Therefore, it can be concluded that WF alone cannot provide internal bond strength to the panels, and the good results obtained with TSSL+WF-RT are a consequence of a synergic combination of TSSL and WF. It was not possible to test a pre-heated WF formulation, because, as discussed before, in the absence of TSSL the starch granules undergo gelatinization and the viscosity increase does not allow efficient mixing with wood particles.

None of the PBs produced with TSSL+WF binder presented water resistance, having disintegrated after immersion in water for 24 h at 20 °C. This makes these PBs unsuitable for use in humid or exterior conditions. Nevertheless, they can be used for interior applications because water resistance is not a requisite for dry condition applications. Moreover, as PB manufacturers use finish foils, melamine-impregnated paper, or high-pressure laminates to overlay PBs, they are protected against moisture to some extent. Chemical cross-linkers, like diisocyanates or dialdehydes, can also be added to the TSSL+WF binder to increase the water resistance.

Finally, as TSSL presents residual contents of sulfur compounds, when producing these particleboards industrially it must be assured that the time-weighted average of SO_2_ in the atmosphere is not higher than 2 ppm [45].

## 4. Conclusions

The present work proposes a low-cost, eco-friendly waterborne adhesive obtained by combining thick spent sulfite liquor (TSSL) with wheat flour (WF). The results showed that TSSL has adhesive properties and it is possible to manufacture particleboards with internal bond strengths (0.30 N∙mm^−2^), above the requirements of standard EN 312 for particleboards type P1 (0.24 N∙mm^−2^). The results were improved when TSSL was combined with WF, particularly when the mixture was pre-heated at 94 °C. This heating treatment increased the binder viscosity, controlling its wood penetration and avoiding starved bond-lines. Additionally, it caused hydrolysis of glycosidic linkages and made the granules more fragile, increasing the number of hydroxyl groups available for interaction with the wood surface. Particleboards with internal bond strength of 0.46 N∙mm^−2^ were obtained, which is above the requirements of standard EN 312 for particleboards type P2 (0.35 N∙mm^−2^). The starch granules present in the wheat flour did not open during the mixture preparation at 94 °C, due to inhibition of gelatinization by TSSL. This allowed the final binder mixture to have sufficiently low viscosity for particleboards production. If starch gelatinization had occurred, mixing of the binder with the wood particles would have been impossible.

The only limitation of the particleboards in terms of performance had to do with lack of water resistance, which makes them usable only for interior applications. Use of chemical cross-linkers would enable this problem to be overcome.

## Figures and Tables

**Table 1:** Final Properties of the thick spent sulfite liquor (TSSL) and TSSL+WF (wheat flour)

Resin	pH	Viscosity (mPa·s)	Solids Content (%)
TSSL	2.79 ± 0.23	115 ± 50	53.7 ± 2.5
TSSL+WF-RT	2.83 ± 0.12	230 ± 15	57.2 ± 1.1
TSSL+WF-94	2.88 ± 0.08	505 ± 19	57.4 ± 0.8

**Table 2:** Assignment of the Infrared Bands of the TSSL Sample [40,41].

Chemical Structure Assignment	Frequency (cm^−1^)
Carbohydrate vibration (C–O, C–C stretching, and C–OH bending)	1037, 1114, and 1155
Syringyl ring breathing with CO stretching	1330
Aromatic skeleton vibration	1429, 1459, 1517, and 1616

**Table 3:** Assignment of the infrared bands of the WF sample [42,43,44].

Chemical Structure Assignment	Frequency (cm^−1^)
Amide I, C=O stretching in gluten protein amides	1650
Amide II, N–H deformation in gluten protein amides	1539
Overlapping effect of N–H in-plane bending, C–N stretching, C–H and N–H deformation vibration of gluten proteins (amide III), and in-plane bending of C–H and C–OH of starch	1454–1207
Asymmetric glucose ring breathing and stretching of C–O–C and C–C bonds in glycosidic linkages	1149
C–OH in-plane bending of starch molecules	1077
In-plane bending of CH_2_ and COH; Stretching of C–O in C–O–C glycosidic linkage	997
In-plane bending of COH and CH of C-1; Symmetrical stretching of C–O–C in glycosidic linkage	928
Symmetrical stretch of C–O–C bond and ring breathing	859
CH_2_ rocking	760
Out-of-plane bending of OH hydrogen bonds	703
